# Somatosensory Cross-Modal Reorganization in Children With Cochlear Implants

**DOI:** 10.3389/fnins.2019.00469

**Published:** 2019-06-26

**Authors:** Garrett Cardon, Anu Sharma

**Affiliations:** ^1^Department of Psychology, Colorado State University, Fort Collins, CO, United States; ^2^Department of Speech, Language, and Hearing Sciences, University of Colorado Boulder, Boulder, CO, United States

**Keywords:** cochlear implants, cross-modal reorganization, somatosensory, vibrotactile, cortical somatosensory evoked potential, high density EEG, independent components analysis, sLORETA

## Abstract

Deprived of sensory input, as in deafness, the brain tends to reorganize. Cross-modal reorganization occurs when cortices associated with deficient sensory modalities are recruited by other, intact senses for processing of the latter’s sensory input. Studies have shown that this type of reorganization may affect outcomes when sensory stimulation is later introduced via intervention devices. One such device is the cochlear implant (CI). Hundreds of thousands of CIs have been fitted on people with hearing impairment worldwide, many of them children. Factors such as age of implantation have proven useful in predicting speech perception outcome with these devices in children. However, a portion of the variance in speech understanding ability remains unexplained. It is possible that the degree of cross-modal reorganization may explain additional variability in listening outcomes. Thus, the current study aimed to examine possible somatosensory cross-modal reorganization of the auditory cortices. To this end we used high density EEG to record cortical responses to vibrotactile stimuli in children with normal hearing (NH) and those with CIs. We first investigated cortical somatosensory evoked potentials (CSEP) in NH children, in order to establish normal patterns of CSEP waveform morphology and sources of cortical activity. We then compared CSEP waveforms and estimations of cortical sources between NH children and those with CIs to assess the degree of somatosensory cross-modal reorganization. Results showed that NH children showed expected patterns of CSEP and current density reconstructions, such that postcentral cortices were activated contralaterally to the side of stimulation. Participants with CIs also showed this pattern of activity. However, in addition, they showed activation of auditory cortical areas in response to somatosensory stimulation. Additionally, certain CSEP waveform components were significantly earlier in the CI group than the children with NH. These results are taken as evidence of cross-modal reorganization by the somatosensory modality in children with CIs. Speech perception in noise scores were negatively associated with CSEP waveform components latencies in the CI group, suggesting that the degree of cross-modal reorganization is related to speech perception outcomes. These findings may have implications for clinical rehabilitation in children with cochlear implants.

## Introduction

Permanent hearing loss in children is a common condition that is found in 2–3 of every 1,000 live births (Centers for Disease Control and Prevention [CDC], 2010). Children who are identified with more severe cases of hearing loss (i.e., ≥70 dBHL), are often candidates for treatment with a cochlear implant (CI). CIs are devices that restore hearing to deaf individuals via direct electrical stimulation of the auditory (VIII) nerve. As of 2013, approximately 324,200 CIs had been fitted worldwide (Food and Drug Administration [FDA], 2012). These devices have proven extremely useful in restoring auditory function to many children born with hearing loss. However, many other implant recipients have had relatively little success in behavioral speech understanding (Harrison et al., 2005; Nicholas and Geers, 2007; Holt and Svirsky, 2008). Despite ongoing improvement in CIs and implantation procedures, there remains a high degree of variability in the behavioral outcomes (e.g., speech and language development) of children with CIs (Svirsky et al., 2000; Sarant et al., 2001, 2014; Tobey et al., 2003; Harrison et al., 2005; Geers, 2006; Nicholas and Geers, 2007; Holt and Svirsky, 2008; Geers et al., 2009; Lund, 2016; Szagun and Schramm, 2016). Given this variability, it is difficult to predict the level of benefit an implant will provide a given patient. Recent investigation has been aimed at discovering the underlying factors associated with this variability (Svirsky et al., 2000; Sarant et al., 2001; Tobey et al., 2003; Geers, 2006; Geers et al., 2009; Szagun and Schramm, 2016). However, despite these efforts, only these factors explain on a portion of the variability (i.e., approximately 35–62%; Fink et al., 2007), though one that seems to stand out is age at implantation – earlier implantation appears to lead to greater chances for favorable outcome (Sharma et al., 2002a,b; Svirsky et al., 2004; Geers, 2006; Geers et al., 2009; Niparko et al., 2010).

Sensory loss (i.e., blindness and deafness) can lead to reorganization of the cerebral cortex. In deafness, this reorganization manifests itself when sensory modalities that are intact recruit auditory cortices for their own processing – termed *cross-modal reorganization* (see Bavelier and Neville, 2002; Merabet and Pascual-Leone, 2010 for reviews). In conjunction with age of implantation, it is likely that cortical development and neuroplastic processes, such as cross-modal reorganization, play a role in outcomes for children with CIs. Though most of the work that characterizes this type of plastic change has examined cross-modal reorganization of auditory cortex by vision (e.g., Rebillard et al., 1977; Neville et al., 1983; Finney et al., 2001, 2003; Fine et al., 2005; Sadato, 2005; Doucet et al., 2006; Bavelier and Hirshorn, 2010; Lomber et al., 2010; Meredith and Lomber, 2011; Campbell and Sharma, 2014, 2016; Clemo et al., 2014; Kok et al., 2014; Scott et al., 2014), a number of studies have also shown evidence of cross-modal reorganization between the somatosensory and auditory cortices in both animals and humans (Levänen et al., 1998; Baldwin, 2002; Auer et al., 2007; Sharma et al., 2007; Allman et al., 2009; Meredith and Lomber, 2011; Meredith and Allman, 2012; Karns et al., 2012). However, while such investigations have been carried out in adults, no study has examined somatosensory cross-modal reorganization of auditory cortical areas in pediatric CI recipients. Thus, the goal of this study was to examine possible cross-modal reorganization between the somatosensory and auditory systems in children with CIs (relative to age-matched NH controls) and its relationship to behavioral speech perception.

## Materials and Methods

### Participants

Participants for the current study consisted of two groups of individuals: those with NH and children with CI. Children with CIs were limited to those with sensorineural hearing loss (SNHL). All participants were recruited and tested in accordance with the Institutional Review Board of the University of Colorado at Boulder. As such, signed informed consent was obtained from parents or guardians of all subjects in the current study. We recruited 35 NH children between the ages of 5 and 17 years of age (17 female). The overall group was divided into three age groups for recruiting and analysis. These groups were: (1) 5–7-year-old children (*n* = 9; mean age = 6.95 years; SD = ±0.53 years); (2) 8–10-year-old children (*n* = 11; mean age = 9.81 years; SD = ±0.97 years); (3) 11-year-old and older children and adolescents (*n* = 15; mean age = 12.9 years; ±SD = 1.45 years). All of these individuals had NH, which was defined as auditory thresholds at or below 20 dBHL at 250, 500, 1,000, 2,000, 4,000, and 8,000 Hz. These thresholds were obtained in each participant by a certified clinical audiologist. Additionally, none of the participants had any history of neurological disorder.

We recruited children with CIs (CI group; *n* = 12; mean age at test = 12.42 years; S.D. = ±4.16 years). A subset of the above NH group was formed for comparison with CI children (NH group; *n* = 17; mean age at test = 12.29 years; S.D. = ±2.46 years). These 17 children were selected, rather than 12, to increase statistical power, while still maintaining similarity in age between the CI and NH groups. Statistical comparison of the ages of the NH and CI groups confirmed that they were not significantly different (*p* > 0.05). Ten out of 12 CI participants were bilaterally implanted, while the remaining two subjects had unilateral CIs. All bilateral CI recipients were implanted sequentially – seven of ten received their first implant in the right ear. The mean age of first implantation for the CI group as a whole was 3.90 years (S.D. = ±4.03 years), while the average age of second implantation for bilaterally implanted children was 7.33 years (S.D. = ±4.47 years). The average duration of first CI use at the time of testing (i.e., time between first CI fitting and testing) was 8.51 years (S.D. = ±4.09 years), while the duration of 2nd CI use was 5.65 years (S.D. = ±2.31 years). Make and model of CI and speech processing strategy was not accounted for given the limited sample size of the CI group.

### Stimuli

250 Hz tones, each 90 ms in duration, with 10 ms linear ramps at onset and offset, were used to elicit cortical somatosensory evoked potentials (CSEP). These stimuli were presented to each participant via a standard clinical bone oscillator (Sensory Systems d.b.a. Radioear Inc., B71 Bone Transducer), which was electrically shielded with copper mesh so that any electrical noise produced by the device would not be registered by the EEG electrodes. During testing, this transducer was temporarily affixed to the participant’s right or left index finger using medical tape. For consistency, all participants underwent testing with right finger stimulation. Additional testing with left finger stimulation was achieved in a subset of the study participants (*n* = 6), though, due to time constraints and subject cooperation, this was not done in all children. Stimulus presentation timing was controlled by E-Prime^®^ 2.0 software (Psychology Software Tools, Inc.). All stimuli were presented at a level of 55 dBHL on the audiometer, which resulted in vibrotactile sensation in all participants (approximately 0.122 g or 1.2 m/s^2^ of acceleration output) that was sufficient to elicit CSEPs, but never uncomfortable (Weinstein, 1968). For all CI participants, CIs were turned off during CSEP recording to ensure that the vibrotactile stimuli were only felt and not heard. Continuous white noise was played via a loudspeaker at a level of 50 dBHL on the side of stimulation in order to mask any auditory artifact of vibrotactile stimulation for all participants. Procedures were similar to those described previously in studies from our laboratory and others (Yamaguchi and Knight, 1991; Sharma et al., 2015, 2016; Cardon and Sharma, 2018). All participants reported that they could *feel*, but not *hear*, the stimulus.

### EEG Recording and Analysis

During testing, each participant was seated in a comfortable chair situated in a sound treated room. They were fitted with a 128-channel EEG recording net (Electrical Geodesics, Inc.) that had been soaked in a solution of water, baby shampoo, and sodium chloride. EEG recordings were sampled at 1 kHz and band-pass filtered online between 0.1 and 200 Hz. Following recording, EEG data were initially high-pass filtered offline at 1 Hz. These data were then segmented into epochs that consisted of 100 ms pre- and 495 ms post-stimulus intervals. Then, data were exported for further analysis in the EEGLAB toolbox (Delorme and Makeig, 2004) running within the Matlab^®^ software package (The MathWorks^®^2014). Once imported, channels containing excessive amounts of noise were rejected. Then, epochs that presented with data exceeding ±100 μV in amplitude were also eliminated. The sampling rate of the data was then changed to 250 Hz to allow for subsequent processing efficiency. Data then underwent re-referencing to a common average reference. Finally, rejected channels’ data were replaced via spherical interpolation, which was necessary to appropriately address highly noisy channels and remove their effects on subsequent analyses.

The region of interest (ROI) employed for initial CSEP analysis in the large NH group consisted of 24 electrodes that covered the parietal and temporal areas of the left hemisphere of the scalp (Hämäläinen et al., 1990). Waveforms from the designated electrodes from this ROI were averaged together to form a composite waveform. Peak latencies and absolute and peak-to-peak amplitudes for the P50, N70, P100, N140a, and N140b CSEP waveform components were then extracted from waveforms from the ROI for each participant. These were later used for statistical comparison.

For CI children (and smaller age-matched group of NH children) electrodes were divided into more specific ROIs in the temporal and parietal regions of both hemispheres in order to evaluate possible effects of cross-modal reorganization on CSEP responses. ROI selection was based on a combination of visual inspection of the 128-channel data and optimal recording locations of CSEPs reported in Hämäläinen et al. (1990) and Cardon and Sharma (2018). ROIs included: (1) Left Temporal ROI (LTemp ROI; electrodes: TP7, T9, P9, TP9, T5-P7); (2) Left Parietal ROI (LPar ROI; electrodes: P3, P5, CP1, P1, PO7, PO3); (3) Right Parietal ROI (RPar ROI; electrodes: P4, P6, CP2, P2, PO8, PO4); (4) Right Temporal ROI (RTemp; electrodes: TP8, T10, P10, TP10, T6-P8). These electrode positions represent approximate 10–20 system electrode locations, as reported in Luu and Ferree (2000), since EGI uses a geodesic electrode organization system.

Average CSEPs were calculated for each participant for all ROIs. Then, each participant’s ROI CSEP waveform component latencies and absolute and peak-to-peak amplitudes were noted (i.e., the difference between the amplitude of the CSEP peak of interest and the preceding peak were calculated). We measured peak-to-peak amplitudes due to the larger inherent variability in measurement of absolute amplitudes (e.g., Johnson et al., 1975). Statistical comparisons were then performed with these CSEP peak latency and amplitude values between the CI and NH groups using non-parametric Mann–Whitney *U* Tests for each ROI. CSEP latencies and amplitudes for the CI group were also correlated (Spearman’s Rank correlations) with behavioral speech perception in noise scores (see “Speech Perception in Noise” section) to evaluate potential relationships between neural activity and behavioral speech perception in noise. Correction for multiple comparisons was performed on both between group statistics and correlations using the False Discovery Rate method presented by Benjamini and Hochberg (1995; *q* ≤ 0.1).

### Current Density Reconstruction

In preparation for current density reconstruction (CDR), each subject’s data epochs were concatenated and subjected to independent components analysis (ICA). One application of ICA is artifact rejection. Thus, independent components (ICs) containing eye blinks or movement, electrical noise, or muscle artifact were removed from each participant’s dataset. After ICA artifact rejection, ICs that accounted for the highest portion of the variance around each peak of the CSEP were saved for inclusion in CDR. Data were then transferred to the Curry^®^ Scan 7 Neuroimaging suite (Compumedics Neuroscan^TM^) for cortical source estimation. Initial processing steps toward CDR included baseline correction, noise estimation using the pre-stimulus interval, averaging of participants individual CSEP waveforms to for grand average waveforms, and additional ICA.

Modeling of the head was accomplished using the Boundary Element Method (BEM; e.g., Fuchs et al., 2002; Hallez et al., 2007). Within this head model, white matter volumes were adjusted to match age-related values (Wilke et al., 2006; Gilley et al., 2008). CDR were then performed for each CSEP waveform component (P50, N70, P100, N140a, N140b) using the sLORETA algorithm (Pascual-Marqui, 2002; see Grech et al., 2008 for a review). The results of this method appear as color gradients that represent the *F*-distribution of the data, which were overlaid using the Montreal Neurologic Institute (MNI) average brain (Evans et al., 1993).

### Speech Perception in Noise

Speech perception ability was assessed in each participant in the CI group using the BKB-SIN^TM^ test (Bench et al., 1979; Etymotic Research, 2005). During this testing, participants sat facing a loudspeaker at 0° azimuth with his or her CI on and functioning as it normally would. Sentences – two lists of six sentences each – were then presented to the participant via the loudspeaker at 65 dBHL. As the sentences progressed, background noise (multi-talker babble) level was increased with each sentence. This noise increase occurred in five steps, each of 5 dB increments, or from 25 dB SNR (least challenging) to 0 dB SNR (most challenging). The participant was asked to repeat the words of the sentence he or she heard. The tester marked key words from each sentence as correct or incorrect and computed a score for each list, based on the number of words repeated correctly. Participants received an SNR score, representing the level at which they could perceive and repeat 50% of key words – lower scores indicated better performance. Scores from the two presented lists were then averaged together to obtain a composite BKB-SIN score for each participant. In addition, age corrections were applied to participants’ composite scores to normalize results for comparison across subjects (Etymotic Research, 2005). Finally, BKB-SIN scores were correlated with CSEP component peak latencies from each ROI to assess the relationship between speech perception in noise and cross-modal reorganization.

## Results

### Normal Hearing Children (*n* = 35)

#### Cortical Somatosensory Evoked Potentials

**FIGURE 1:**
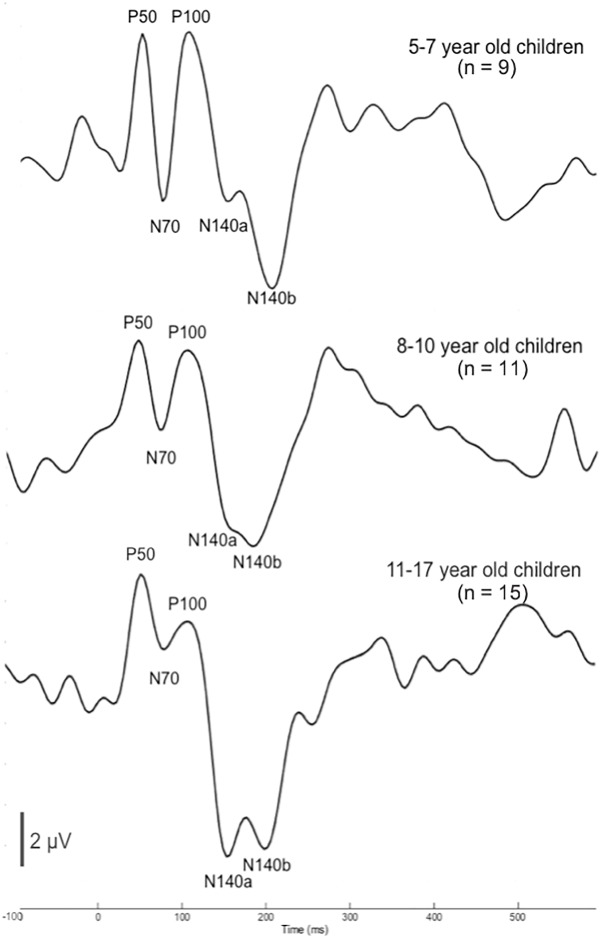
Cortical somatosensory evoked potentials (CSEP) waveforms for children 5–7 (*n* = 9), 8–10 (*n* = 11), and 11–17 (*n* = 15) years old show consistent morphology across age. CSEP waveforms from the temporo-parietal ROI for each of the three age groups of children with normal hearing in the study: (1) 5–7-year-old children (top); (2) 8–10-year-old children (middle); (3) 11–17-year-old children (bottom). Each waveform shows all CSEP waveform components of interest – P50, N70, P100, N140a, and N140b.

Plots of the grand average CSEP waveforms for each of the age groups (i.e., 5–7-, 8–1-, and 11–17-year-olds) from the temporo-parietal ROI are shown in Figure 1. Across all ages, all of the components of the CSEP (i.e., P50, N70, P100, N140) can be reliably identified. In the majority of subjects, regardless of their age, the N140 appeared as a bifid negative going peak. Given this pattern, we classified the first of the N140 peaks as the N140a, while the second was called the N140b. Thus, CSEP waveform morphology appears to be stable (with respect to presence of peak components) across the age range examined in this study.

In order to determine more detailed differences between the age groups’ CSEP waveforms, both peak latency and peak-to-peak amplitude results from the aforementioned ROI were subjected to statistical comparison. One latency difference was found following multiple comparisons correction. That is, there was a main effect of age for the N140a CSEP latency (*p* = 0.00; *F* = 8.05). *Post hoc* analysis revealed that the youngest group (5–7-year-old) showed significantly shorter latencies compared with the 8–10-year-old group for the latency of the N140a CSEP peak (*p* = 0.00). The 5–7-year-old children also exhibited significantly larger CSEP peak-to-peak amplitudes for the N70 (*p* = 0.003; *F* = 7.26), P100 (*p* = 0.004; *F* = 6.66), and N140b (*p* = 0.002; *F* = 7.483) CSEP components relative to the two older groups. The latency finding is reflective of expected developmental patterns and consistent with previous studies (e.g., Allison et al., 1984; Sitzoglou and Fotiou, 1985; Pihko et al., 2009). However, no previous studies have reported on the maturation of amplitude of CSEPs recorded to vibrotactile stimuli in the literature possibly reflecting the inherent variability in absolute amplitude measurements.

#### Current Density Reconstructions

**FIGURE 2:**
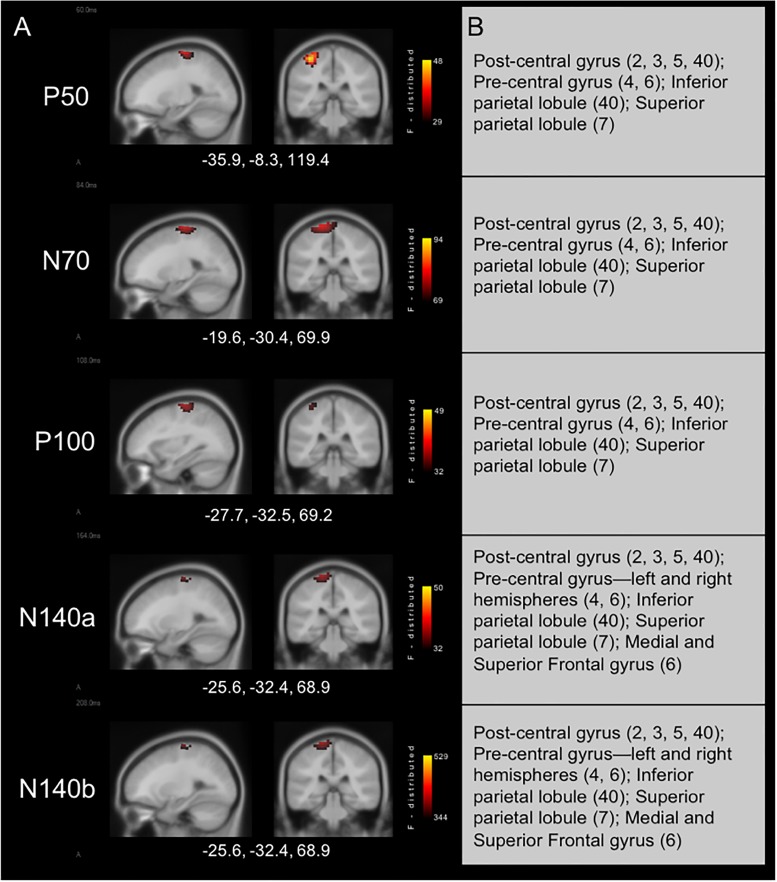
Current density reconstructions for cortical somatosensory evoked potentials (CSEP) waveform components in children with normal hearing (NH) show expected activation of parietal cortices contralateral to the side of stimulation. **(A)** Cortical activations in response to vibrotactile stimulation of the right index finger in children with NH. Activations are organized in rows corresponding to each CSEP waveform component. Sagittal (left) and coronal (right) slices are presented for each of these components. Three-dimensional Montreal Neurological Institute coordinates for each activation are listed below each row of slices. The *F*-distribution scale presents the color gradient associated with the maximum (yellow) through the minimum (black) likelihood for activation as calculated by sLORETA. **(B)** A table listing all areas of significant activation for each CSEP waveform component. Brodmann areas are indicated in parentheses.

Results from cortical source localization analysis for NH children (*n* = 35) are shown in Figure 2. Initially, sources were computed for each age group separately. However, it was found that all groups’ source estimations were comparable. Thus, all participants were combined for final cortical source analysis. Visual inspection and computer-aided determination of the areas of significant activation yielded by sLORETA analysis revealed the following: (1) the P50, N70, and P100 CSEP waveform components presented with virtually the same areas of activation of the left hemisphere. These included, post-central gyrus (BA 2, 3, 5, 40), pre-central gyrus (BA 4, 6), inferior parietal lobule (BA 40), and superior parietal lobule (BA 7); 2) the N140a and N140b generators were also very similar. In addition to all of the previously mentioned activated areas (i.e., for the P50-P100 CSEP components), medial and superior frontal gyri were also activated for the N140a and N140b.

Due to the constancy in peak CSEP components and CDR across the 5–17-year-old age range found in the NH group (*n* = 35) and to increase power for this study, we grouped all CI participants’ CSEP data for analysis and comparison against a subset of age-matched NH children (*n* = 17).

### Cochlear Implanted Children (*n* = 12) and Age-Matched NH Children (*n* = 17)

#### Cortical Somatosensory Evoked Potentials

**FIGURE 3:**
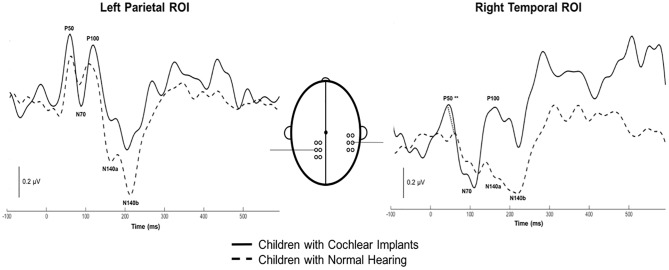
Grand average cortical somatosensory evoked potentials (CSEP) waveforms from the LPar and RTemp ROIs for both the CI (solid lines) and NH (dashed lines) groups. The latency of the P50 CSEP waveform component from the RTemp ROI was significantly earlier in the CI participants than the NH group (*p* = 0.00; *U* = 177.00; indicated by double asterisks).

Both CI and NH children presented with CSEP waveform morphology that was typical of somatosensory evoked responses elicited via vibrotactile stimulation of the finger (Hämäläinen et al., 1990), especially in the parietal ROI contralateral to the side of stimulation. Figure 3 (left panel) shows grand average results for both groups of children from the LPar ROI during right index finger stimulation. Both groups’ results show the characteristic CSEP waveform peaks – P50, N70, P100, N140a, and N140b. While there were no significant differences found in the latencies and amplitudes of the CSEP peaks from this ROI, it is shown here for reference. In contrast, the RTemp ROI waveforms showed a significant difference between the latency of the P50 CSEP component between groups (Figure 3, right panel; *p* = 0.00; *U* = 177.00) (see also Table 1), such that the CI group’s latencies were significantly earlier (mean = 49.00 ms; S.D. = 7.5 ms) than the NH group (mean = 65.18 ms; S.D. = 13.17 ms). Additionally, the morphology of the CSEP waveforms differed somewhat between groups and ROIs. For instance, the CI group’s grand average waveform shows more robust peaks than those of the NH group. Additionally, the morphology of the CI group’s waveform includes a small positivity at approximately 50 ms, followed by a large negativity around 100 ms, and then another positivity between 150 and 200 ms. This waveform morphology pattern may be more characteristic of the cortical auditory evoked potential, as observed in older children, than the CSEP (e.g., Sharma et al., 1997; Gilley et al., 2005). A shorter P50 latency in the RTemp ROI of the CI children, in response to vibrotactile stimulation, may be a marker of cross-modal reorganization of temporal cortices by the somatosensory system. In addition, somatosensory evoked potentials originating from the auditory cortices may maintain some aspects of auditory evoked potential morphology.

**Table 1:** Mean latency, standard deviation, 95% confidence intervals, and significance statistics for all CSEP latencies from the LPar and RTemp ROIs.

ROI and Waveform Component	Group	Mean Latency (ms)	Std. Deviation	95% Confidence Interval (Upper – Lower Bound)	*p*-value; Mann–Whitney *U* statistic
LPar P50	CI	59.4	7.3	54.8 – 64.1	0.39; 121.5
	NH	61.7	6.9	58.1 – 65.3	
LPar N70	CI	87.3	9.6	81.2 – 93.4	0.19; 72.0
	NH	82.8	8.3	78.5 – 87.1	
LPar P100	CI	117.3	14.3	108.2 – 126.4	0.37; 81.0
	NH	112.9	14.1	105.7 – 120.2	
LPar N140a	CI	152.0	25.3	135.9 – 168.0	0.21; 131.0
	NH	161.9	18.2	152.5 – 171.3	
LPar N140b	CI	204.7	28.7	186.4 – 222.9	0.59; 114.5
	NH	215.3	22.5	203.7 – 226.8	
**^∗∗^RTemp P50L**	**CI**	**49.0**	**7.5**	**44.3** – **53.7**	**0.00; 177.0**
	**NH**	**65.2**	**13.2**	**58.4** – **71.9**	
RTemp N70L	CI	83.3	17.5	72.2 – 94.4	0.15; 135.0
	NH	94.6	15.9	86.4 – 102.7	
RTemp P100L	CI	122.7	24.8	106.9 – 138.4	0.37; 81.5
	NH	113.6	18.7	104.0 – 123.3	
RTemp N140a	CI	148.3	24.9	132.5 – 164.2	0.40; 82.0
	NH	141.4	20.6	130.8 – 151.9	
RTemp N140b	CI	216.0	26.1	199.4 – 232.6	0.36; 81.5
	NH	204.5	25.7	191.2 – 217.7	

*Bold type indicates a significant difference between the CI and NH groups. ^∗∗^p < 0.00.*

#### Current Density Reconstructions

**FIGURE 4:**
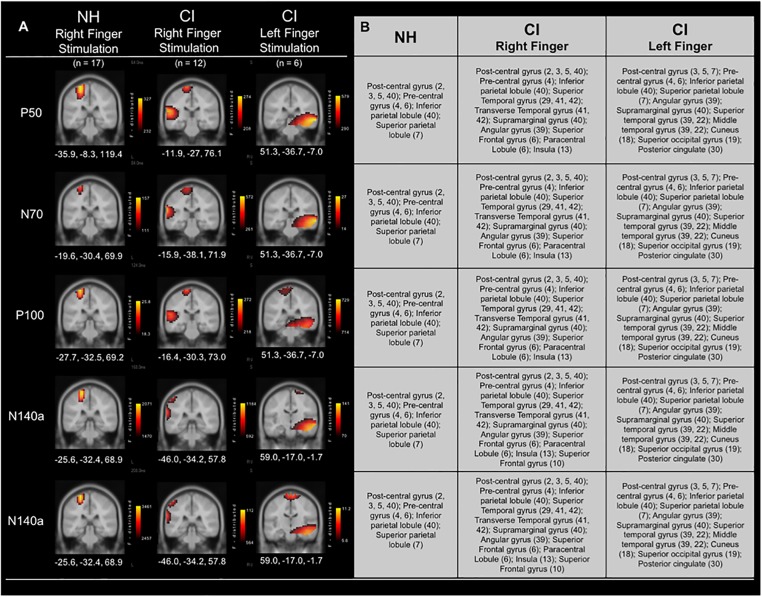
Current density reconstructions (CDR) for cortical somatosensory evoked potentials (CSEP) in normal hearing and cochlear-implanted children. **(A)** Cortical activations in response to vibrotactile stimulation of the right index finger in children with normal hearing (NH – left panel; *n* = 35) and cochlear implants (CI – middle panel; *n* = 12). Additionally, cortical activations in response to stimulation of the left finger in a subset of CI children who received their initial CIs in the right ear are shown in the right-most panel (*n* = 6). Activations are organized in rows corresponding to each CSEP waveform component (P50, N70, P100, N140a, N140b). CDRs are presented on coronal slices for each of these components. Three-dimensional Montreal Neurological Institute coordinates for each activation are listed below each MRI slice. The *F*-distribution scale (bottom) presents the color gradient associated with the maximum (yellow) through the minimum (black) likelihood for activation as calculated by sLORETA. **(B)** A table listing all areas of significant activation for each CSEP waveform component. Brodmann areas are indicated in parentheses.

Current Density Reconstructions were performed for each of the CSEP waveform components. Figure 4 shows CDR results for vibrotactile stimulation of the right finger in both the CI group and NH group. In addition, cortical activations in response to left finger stimulation in a subgroup of CI children who received their first implant on the right side are shown in Figure 4. In response to vibrotactile stimulation of the right finger (Figure 4 – middle panel), CI children, as a group, show clear activation of the left (i.e., contralateral to the side of stimulation) somatosensory cortices (i.e., post-central gyrus; BA 3, 2, 5), as well as pre-central gyrus (BA 4, 6), inferior and superior parietal lobules (BA 40 and 7), respectively. Contralateral activations in these areas were expected (i.e., due to the crossover of ascending somatosensory pathways) and consistent with those calculated for the NH group (Figure 4 – left panel). However, the CI group also showed robust activation of the left temporal cortex – superior temporal gyrus (BA 29, 41, 42); transverse temporal gyrus (BA 41, 42); supramarginal gyrus (BA 40); Angular gyrus (BA 39); superior frontal gyrus (BA 6); paracentral lobule (BA 6); and insula (BA 13). This pattern of activation was consistent for the P50, N70, and P100 CSEP waveform components (Figure 3). Both the N140a and N140b presented with CDRs that matched the above CSEP components. However, in addition, frontal cortices contributed to these later components in the CI group. CDR analysis showed that another portion of the superior frontal gyrus contributed to these components (i.e., BA 10; see Figure 3)

In a subset of CI participants (*n* = 6), the left index finger was stimulated in addition to the right (separate conditions). All of these children received implants in their right ears first (mean age at first implantation = 2.89 years; S.D. = ±2.67 years). Five out of six of these participants were also implanted in the left ear at a later date (mean age at second implantation = 7.47 years; S.D. = ±2.91 years). Figure 4 (right panel) shows the CDR for the right and left index finger stimulation in these children. Interestingly, the cortical activations to the left finger stimulation in the subgroup of children who had received their first CI in the right ear appeared to be centered primarily in auditory cortical areas, with some activity evident in known somatosensory cortical regions. These activated areas included: Superior temporal gyrus (39, 22); Middle temporal gyrus (39, 22); Post-central gyrus (3, 5, 7); Pre-central gyrus (4, 6); Inferior parietal lobule (40); Superior parietal lobule (7); Angular gyrus (39); Supramarginal gyrus (40). These areas of activation were largely found in the right hemisphere, though in the P100 and N140b CSEP components, post-central gyrus (i.e., somatosensory cortex) activations were partially located in the left hemisphere. Activation of auditory processing areas (BA 39, 22) in response to vibrotactile stimulation of the left finger suggests additional cross-modal reorganization of the auditory cortex ipsilateral to the side of first implantation.

#### CSEP Correlation With Speech Perception in Noise

**FIGURE 5:**
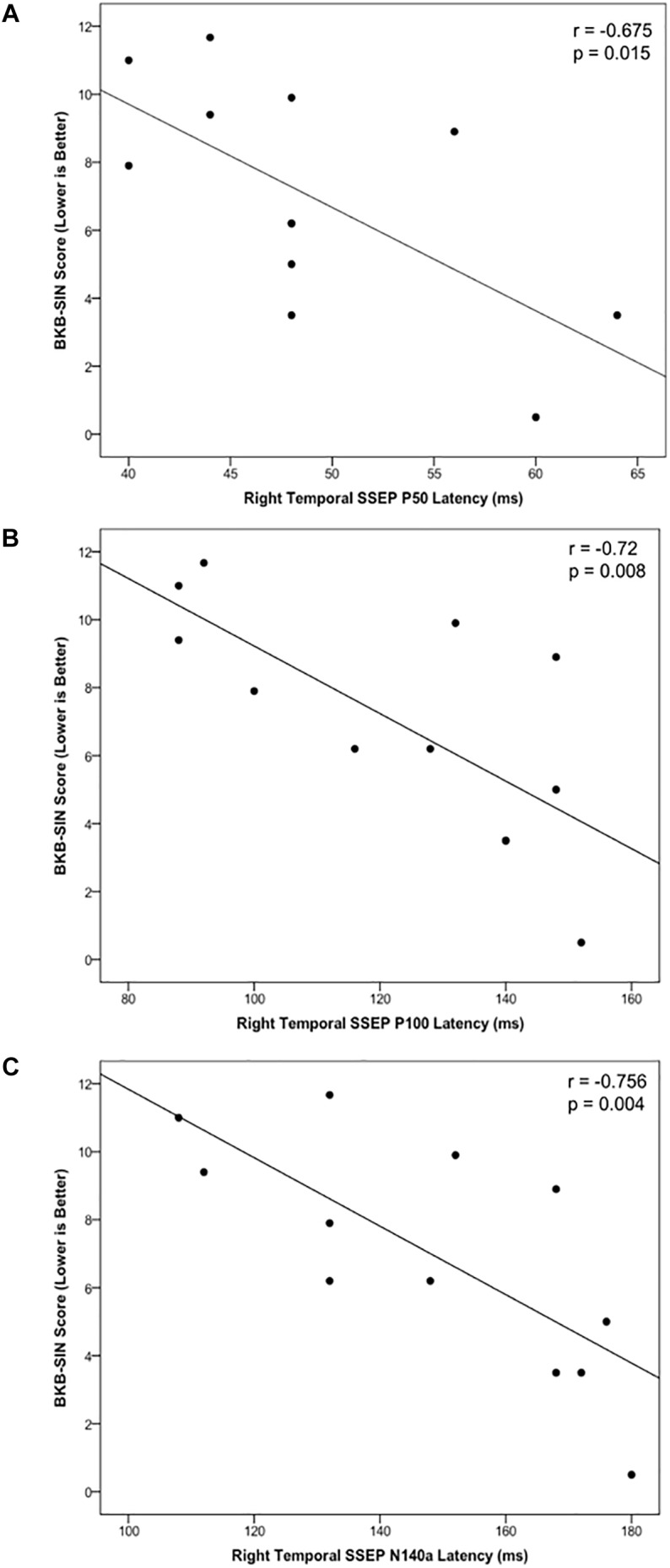
Scatter plots describing significant relationships between scores on the BKB-SIN and the CSEP P50 **(A)**, P100 **(B)**, and N140a **(C)** waveform components in the CI group.

Cortical somatosensory evoked potentials peak measurements from the LTemp, LPar, RPar, and RTemp ROIs were correlated with results on the BKB-SIN for the CI group to assess the relationship between neurophysiological activity and behavioral speech perception in noise. The latencies of the P50, P100, and N140a from the RTemp ROI all showed significant negative correlations with BKB-SIN score (Figure 5A–C; *r* = –0.679, *p* = 0.015; *r* = –0.72, *p* = 0.008; *r* = –0.756, *p* = 0.004, respectively). That is, as latency decreased, BKB-SIN score worsened (see Figure 5). That decreased behavioral performance was related to earlier CSEP latencies from the right temporal region of the scalp may suggest that children who have trouble with speech perception in noise, show more evidence of cross-modal reorganization consistent with previous studies (Doucet et al., 2006; Sandmann et al., 2012; Campbell and Sharma, 2016).

## Discussion

The objective of the current study was to determine whether cochlear-implanted children would show evidence of cross-modal reorganization of the auditory cortex by the somatosensory system, and if this reorganization would be correlated with behavioral outcomes in these children. Using high-density EEG recorded in response to vibrotactile stimulation of the right and left index finger, we found the following main results: (i) NH children showed expected small age-related variations in CSEP waveform component latencies between 5–7 and 8–10 years of age. However, the generators of cortical somatosensory activation localized to the post-central gyrus, association cortices of the parietal lobe, and pre-central gyrus contralateral to the side of stimulation across the age span; (ii) CSEP morphology and latencies were consistent between CI and NH children in the LPar ROI, but not the RTemp ROI – the latter exhibiting significantly earlier CSEP latencies in the CI group, (iii) CDR of right finger vibrotactile stimulation revealed expected activation of the left somatosensory cortices in both NH and CI children. However, CI participants showed activation of auditory processing areas in the left temporal and parietal association cortex by vibrotactile stimulation; (iv) In a subset of children who received their first CIs in the right ear, we saw significant cross-modal activation in the right hemisphere, suggesting that the cortex ipsilateral to the first CI (i.e., the cortex less activated by the first implant) is highly susceptible to cross-modal activation; (v) CSEP latencies from the RTemp ROI were significantly correlated with speech perception in noise results, which may be an indication that poorer behavioral outcomes with the CI are associated with greater degrees of cross-modal reorganization.

### CSEP Development in Typical Children

The morphological aspects of the participants’ CSEP data (Figure 1) were consistent with previous reports. For instance, one study (Hämäläinen et al., 1990) used vibrotactile stimuli applied to the middle finger to evoke potentials from the primary and secondary somatosensory cortex and showed P50, N70, P100, and N140 CSEP components. The CSEP waveforms in the current study consistently showed all peak components across the age range. This pattern of stability of peak components across age differs from the developmental progression of cortical evoked potentials recorded to visual and auditory stimuli, which changes significantly throughout the age range studied here (e.g., Placzek et al., 1985; Ellingson, 1986; Ponton et al., 2000; Gilley et al., 2005). In fact, we have observed that the CSEP waveforms of adults (ranging in age from 21 to 71) are morphologically comparable to the CSEP waveforms presented in the current study (Cardon and Sharma, 2018). Thus, it appears that the CSEP may be unique among modalities, in that major peak components are present and remain constant from school age through adulthood. More significant changes in the CSEP waveform likely occur earlier in life (i.e., by age 4) and afterward slow considerably (e.g., Desmedt et al., 1976; Allison et al., 1984; Sitzoglou and Fotiou, 1985; Fagan et al., 1987; Eggermont, 1988; Pihko et al., 2009). Our data seem to support the above notion in that any differences that were seen in amplitude or latency of CSEP components were noted between the youngest and two older groups. These findings may exhibit the ending of the early childhood phase of development of the CSEP (i.e., slowing after age 4 years). Given the age range of the current study, the participants may have been too mature for observation of more robust developmental effects.

Current density reconstructions yielded results that matched both our hypothesis and previously reported findings. Numerous investigations have outlined the generators of the various CSEP components. For instance, previous studies have found that the P50 CSEP component is generated in the post-central gyrus of the cerebral hemisphere contralateral to the side of stimulation in primary somatosensory cortex (SI; e.g., Mauguière et al., 1983). The N70 also appears to be generated in contralateral SI (Michie et al., 1987). Hämäläinen et al. (1990) proposed, based on both animal and human studies (e.g., Hari et al., 1983, 1984; Hämäläinen et al., 1990), that the P100 originates from a combination of ipsi- and contralateral SII cortex. The N140 CSEP component seems to have a number of generators, which are likely distributed throughout the posterior parietal regions of the cortex, with the strongest contributions coming from cortices contralateral to the stimuli. Specifically, some have proposed that the N140 is influenced by generators in contralateral SII (Hari et al., 1983, 1984, 1993) and also contains activity from Brodmann are 46 and other frontal cortices (Desmedt and Tomberg, 1989; Hämäläinen et al., 1990). The current results mirror these reports’ descriptions of the sources of cortical activity that contribute to the CSEP. That is, all CSEP components from the current study were localized to the primary, secondary, and association somatosensory cortices (BA 3, 2, 1, 5, and 7) in the hemisphere contralateral to the side of stimulation. In addition, pre-central gyrus was activated in the CDRs for each of the CSEP components. This activity may be mediated by connections between the pre- and post-central gyrus (e.g., Pandya and Kuypers, 1969). Finally, it may be interesting to note that the N140a and N140b CSEP components show activation of medial and superior frontal cortices (i.e., Brodmann area 6), which is consistent with the characterization of the generators for these components offered by Hämäläinen et al. (1990) that indicate frontal cortex involvement in the generation of the N140 CSEP.

### Evidence and Possible Mechanisms of Somatosensory Cross-Modal Reorganization in CI Children

In CI children, we saw at least two types of evidence for somatosensory cross-modal reorganization: earlier CSEP latencies in the RTemp ROI and activation in auditory processing areas in superior and transverse temporal cortices, as well as cortical regions important to language processing (i.e., parts of Wernicke’s area), in response to somatosensory stimuli (see Figure 1, 2). A number of previous studies have reported similar findings in both animals and humans (Neville and Lawson, 1987; Levänen et al., 1998; Baldwin, 2002; Auer et al., 2007; Sharma et al., 2007; Shore et al., 2008; Karns et al., 2012; Sandmann et al., 2012; Campbell and Sharma, 2014, 2016; Sharma and Glick, 2016). For instance, a recent study from our lab showed a similar pattern of earlier latencies of cortical visual evoked potentials, as well as activation of auditory processing areas in response to visual stimuli, in CI children (Campbell and Sharma, 2016). In contrast, one study in the literature appears to present conflicting evidence to the present results. That is, Hickok et al. (1997) used MEG to study possible cross-modal reorganization in one deaf young adult. These investigators reported that they found no evidence of somatosensory-to-auditory cross-modal reorganization in this subject. However, these investigators used a tapping stimulus applied to the finger, instead of a vibrotactile stimulus. Because of the similarity between sound and vibration, the auditory cortex may be better suited to process vibrotactile input, while this may not be the case with other types of stimuli (i.e., tapping). Additionally, this study only assessed these factors in one subject. Thus, the Hickok et al. (1997) study may not be directly comparable to this, and other, studies that do show evidence of somatosensory cross-modal reorganization. Overall, the majority of studies in the literature submit that cross-modal reorganization of the auditory cortex by the somatosensory system can occur in deaf individuals. We add our findings as another piece of converging evidence that supports this notion in CI children. Future studies should endeavor to replicate these initial findings given the limited sample of CI children in the current study.

The current CI participants presented with robust activity in response to vibrotactile stimuli in primary and secondary auditory cortices, as well as supramarginal and angular gyri, which make up part of Wernicke’s area, important in receptive language processing. Such findings were not the case in NH participants, despite the presence of continuous auditory masking noise. While some have shown cross-modal reorganization primarily in higher order auditory cortices in deaf individuals (Kral, 2007), there is a precedent for primary auditory cortical reorganization. That is, Auer et al. (2007) presented evidence of activity arising from primary auditory cortices in response to vibrotactile stimulation in six deaf young adults using fMRI. Additionally, MEG source analysis performed by Levänen et al. (1998) showed bilateral activation of superior temporal gyrus (STG) in one adult with congenital deafness. It is possible that normally unisensory areas are taken over by other sensory modalities (Auer et al., 2007). Numerous studies have established a precedent for both intracortical, thalamocortical, and subcortical anatomical (e.g., Foxe et al., 2000; Schroeder et al., 2001; Gobbelé et al., 2003; Kayser et al., 2005; Caetano and Jousmäki, 2006; Hackett et al., 2007), as well as functional (Jousmäki and Hari, 1998; Lakatos et al., 2007; Brett-Green et al., 2008), connections between the somatosensory and auditory systems. Subcortically driven cross-modal reorganization of the primary and secondary auditory cortices appears to be a distinct possibility, especially in congenitally deafened individuals whose deprivation was a factor during the development of subcortical-cortical pathways (Soto-Faraco and Deco, 2009; Zeng et al., 2012). These findings are also in agreement with previous data from MEG recordings performed by our group (Sharma et al., 2007), which showed auditory and multimodal association (i.e., Wernicke’s area) activity in response to vibrotactile stimulation of the hands in one deaf adult. In addition to subcortical contributions, given the multimodal nature of these areas, it is possible that unmasking and enhancement of latent multisensory connections when one modality is deprived may contribute to cross-modal reorganization in these cortical regions (Levänen et al., 1998; Auer et al., 2007). Such enhancement could lead to both shorter CSEP latencies – via improved synaptic efficiency – and cross-modal activation.

It may be interesting to note that in all of the previous studies examining cross-modal reorganization in deaf individuals, the duration of deafness was extensive (i.e., into adulthood). For example, the subject recruited for study in Levänen et al. (1998) was 77 years of age and had been deaf for all or most of his life. Though the duration of deafness in the current participants was lower than many of the previous studies – the average age of implantation of children in the current study was 3.9 years – it was beyond the sensitive period for auditory cortical development (i.e., 3.5 years; Sharma et al., 2002a,b). Given that many more children receive their implants around the FDA approved age of 1 year currently, future studies should investigate cross-modal reorganization in children who were fitted with CIs at early ages in order to determine if cross-modal reorganization takes place when the duration of deafness is very short in childhood (see Meredith and Allman, 2012).

### Bilateral Implantation and Somatosensory Cross-Modal Reorganization

In the current results, children who received their CIs in the right ear first and who later received a second CI in the left ear showed differing patterns of cortical activation between the right and left cortical hemispheres in response to somatosensory stimulation of the right and left index fingers. Stimulation of the right index finger lead to activity patterns that, for the most part, were consistent with typical somatosensory responses (post- and pre-central gyri, BA 3, 5; and 4, respectively) and activation of auditory areas (BA 39, 22; consistent with our overall finding of cross-modal recruitment for the CI group as a whole). Results from the stimulation of the left finger were, however, quite distinct. That is, instead of the most robust activations being localized to pre- and post-central gyri, cortical generators were estimated to be in the right temporal areas, especially for the P50 and N70 CSEP components. This finding is suggestive of a higher degree of cross-modal reorganization. Our results agree with the results of a study performed by Kral et al. (2002) in congenitally deaf white cats. These investigators reported that cats who had received their implants late (i.e., >5 months) showed decreased activations in the auditory cortex ipsilateral to the implanted ear, while responses coming from the contralateral auditory cortex did not show the same pattern. Additionally, Gordon and Papsin (2009) reported that longer durations of unilateral CI use in humans (i.e., >2 years) lead to abnormally high lateralization of EEG signals to the auditory cortex contralateral to the CI. In contrast, the auditory cortex ipsilateral to the implant showed very low activation (Gordon et al., 2013). The participants who received their CI in the right ear first were fitted with their first implant around the age of 2.89 years (±2.67 years), which is under the sensitive period for auditory cortical maturation (i.e., 3.5 years) reported by Sharma et al. (2002a,b). Consistent stimulation of the left auditory cortex via a CI placed in the right ear during the sensitive period may have contributed to the results from right finger stimulation that suggest near normal somatosensory activation in children who received their first CI in the right ear and some activation of auditory areas (Figure 4, right panel). In contrast to right finger stimulation, left finger stimulation lead to robust activation of right auditory cortices in these children (Figure 4, left panel) suggesting that the “weaker,” ipsilateral cortex is highly amenable to cross-modal recruitment by the somatosensory modality. Overall, these children spent years without optimal auditory input to the right auditory cortices, which may have allowed cross-modal reorganization of these cortical areas in the cortex ipsilateral to the CI (e.g., Kral et al., 2013; Jiwani et al., 2016). Unfortunately, the present sample of bilaterally implanted children in which left finger stimulation was performed only amounted to six participants. Thus, the above results should be interpreted with caution. Additional studies should be carried out to further investigate the potential effects of unilateral cochlear implantation and hemispheric differences in cross-modal reorganization.

### Connections Between Somatosensory Cross-Modal Reorganization in CI Children and Speech Perception

The current findings suggest a relationship between somatosensory cross-modal reorganization and speech perception in noise in CI children. This relationship was such that children who had poorer speech perception in noise with their implant showed more cross-modal re-organization. This suggests that these individuals may have been activating the somatosensory system to help disambiguate the impoverished signal input from the CI. There are numerous reports in the literature that support the notion of the somatosensory system being involved in speech perception (Liberman et al., 1967; Liberman and Mattingly, 1985; Fadiga et al., 2002; Watkins et al., 2003; Galantucci et al., 2006; Meister et al., 2007; Skipper et al., 2007; Ito et al., 2009; Russo et al., 2012; Ammirante et al., 2013; Hubka et al., 2015). For example, Gick and Derrick (2009) tested NH participants’ phoneme perception (e.g., “p” vs. “b”) while simultaneously presenting inaudible puffs of air to their skin. Interestingly, these participants more often perceived a phoneme as being aspirated when the air puff was presented, reflecting speech-related auditory-tactile integration. Deaf individuals have also shown evidence that they differentiate same-sex talkers and musical instruments solely by using vibrotactile information (Russo et al., 2012; Ammirante et al., 2013). These abilities suggest that the somatosensory system can decipher information that is highly relevant to speech perception, such as frequency and timbre. Furthermore, Ito et al. (2009) showed evidence that stretching the facial skin affected the perception of an auditory phoneme. They reasoned that, since the somatosensory receptors responsible for stretching and orientation of the skin are constantly and systematically being activated during speech *production*, somatosensory input may also be a vital part of speech *perception*. Animal studies have also presented evidence that the somatosensory system may be involved in vocalization behavior. For instance, Hubka et al. (2015) showed that vocalizations in deaf cats may be (partially) influenced by an auditory feedback loop that is mediated by somatosensory perception. These findings are paralleled by studies that have demonstrated that the motor cortices thought to be related to speech production may be activated during speech perception (Fadiga et al., 2002; Watkins et al., 2003; Meister et al., 2007). Thus, it is reasonable to believe that CI users may rely on vibrotactile input to improve understanding (Gick and Derrick, 2009; Huang et al., 2017), especially under challenging listening conditions, such as speech presented in background noise. As such, future research efforts should be devoted to exploring the potential benefits of tactile stimulation for aiding CI users (Huang et al., 2017).

## Conclusion

The current study examined cross-modal reorganization between the somatosensory and auditory systems in children with CIs. CDRs secondary to stimulation of the right index finger revealed cortical activation in somatosensory cortices in both NH and CI groups, while the CI group also presented with cortical activity localized to auditory cortical areas suggestive of cross-moral re-organization. Our results also suggest that the cortex ipsilateral to the first implanted ear (which receives weaker auditory input than the contralateral cortex) is highly susceptible to cross-modal reorganization. Finally, children who have difficulty perceiving speech with the CI are more likely to show cross-modal re-organization, likely as a compensatory adaptation.

## Data Availability

All datasets analyzed for this study are included in the manuscript and the supplementary files.

## Ethics Statement

This study was carried out in accordance with the recommendations of the Belmont Report as reviewed by the Institutional Review Board of the University of Colorado Boulder with written informed consent from all subjects or their guardians. Additionally, all children aged seven and above provided written assent prior to participating in the study. All subjects gave written informed consent/assent in accordance with the Declaration of Helsinki. The protocol was approved by the Institutional Review Board of the University of Colorado Boulder.

## Author Contributions

Both authors contributed equally to the conceptualization, hypothesis development, recruitment, data acquisition, data analysis and interpretation, writing, and editing of the manuscript.

## Conflict of Interest Statement

The authors declare that the research was conducted in the absence of any commercial or financial relationships that could be construed as a potential conflict of interest.
